# Using a Naturalistic Store Laboratory for Clinical Trials of Point-of-Sale Nutrition Policies and Interventions: A Feasibility and Validation Study

**DOI:** 10.3390/ijerph18168764

**Published:** 2021-08-19

**Authors:** Marissa G. Hall, Isabella C. A. Higgins, Anna H. Grummon, Allison J. Lazard, Carmen E. Prestemon, Jennifer Mendel Sheldon, Lindsey Smith Taillie

**Affiliations:** 1 Department of Health Behavior, Gillings School of Global Public Health, University of North Carolina at Chapel Hill, Chapel Hill, NC 27599, USA; ihiggins@email.unc.edu; 2 Lineberger Comprehensive Cancer Center, University of North Carolina at Chapel Hill, Chapel Hill, NC 27514, USA; lazard@unc.edu (A.J.L.); j.mendel.sheldon@unc.edu (J.M.S.); 3 Carolina Population Center, University of North Carolina at Chapel Hill, Chapel Hill, NC 27516, USA; cepreste@live.unc.edu (C.E.P.); taillie@unc.edu (L.S.T.); 4 Harvard Center for Population and Development Studies, Harvard TH Chan School of Public Health, Cambridge, MA 02138, USA; agrummon@hsph.harvard.edu; 5 Department of Population Medicine, Harvard Medical School, Harvard Pilgrim Health Care Institute, Boston, MA 02215, USA; 6 Hussman School of Journalism and Media, University of North Carolina at Chapel Hill, Chapel Hill, NC 27599, USA; 7 Department of Nutrition, Gillings School of Global Public Health, University of North Carolina at Chapel Hill, Chapel Hill, NC 27599, USA

**Keywords:** point of purchase, retail environment, methodology, validation, Latinx health

## Abstract

Point-of-sale policies such as warnings and taxes are promising tools for improving the nutritional quality of food purchases. Research studies conducted in naturalistic store laboratories could improve the quality of evidence about point-of-sale interventions by allowing for realistic exposure in a controlled setting. This study aimed to assess whether purchasing behavior in a naturalistic store laboratory setting was similar to real-life purchasing behavior and to evaluate participants’ perceptions of store realism and the acceptability of research study protocols in this setting. In a longitudinal observational study in 2019, Latinx parents in North Carolina (*n* = 61) attended five weekly visits at the UNC Mini Mart, a naturalistic store laboratory that resembled a small convenience store. At each visit, participants purchased a week’s supply of beverages. Purchases of beverages in the Mini Mart were compared to participants’ purchases from receipts submitted the week prior to the study. Analyses compared the percentage of participants buying sugary drinks and non-sugary drinks in the Mini Mart vs. in real stores using Chi-Square tests with Fisher’s *p*. The percentage of parents who purchased sugary drinks in the Mini Mart (93%) was not significantly different from the percentage who purchased sugary drinks during the week before the study (74%, *p* = 0.28). The percentage purchasing non-sugary drinks was similar in the two settings (85% in the Mini Mart vs. 85% from receipts, *p* = 0.33). Nearly all participants reported that their Mini Mart purchases were similar to real-life purchases (96%); the Mini Mart felt like a real store (94%); they could find all the beverages they were looking for (92%); and they could imagine doing their real-life beverage shopping in the Mini Mart (92%). Moreover, retention was high, with 97% of participants attending the final study visit. These results indicate that naturalistic store laboratories are a promising method for increasing the ecological validity of trials to evaluate point-of-sale interventions.

## 1. Introduction

Poor quality diets, including high consumption of sugary drinks and ultra-processed food, are a major contributor to morbidity and mortality [1,2,3]. Solutions to encourage healthier food purchases in retail settings are urgently needed. Nutrition-related point-of-sale policies and interventions that have shown promise include excise taxes on sugary drinks and ultra-processed food [4,5,6,7]; marketing restrictions [8,9]; and requiring warning labels on sugary drinks and ultra-processed food [9,10,11,12,12]. Most evidence on these policies comes from either real-world observational studies or laboratory-based experimental studies. Observational studies tend to have high external validity but may be limited in their ability to eliminate alternative explanations for observed changes [13]. By contrast, experiments can allow for stronger causal inference but often occur in artificial settings and rely on self-reported outcomes, which may limit their generalizability to real-world settings and behaviors [13]. For example, prior experiments of nutrition interventions have typically involved showing participants a food or beverage product via an online survey, after which participants rate their perceptions of the product or their intentions to purchase the product [10,14,15,16]. Online experiments have several benefits, including that they allow for manipulation of many experimental arms within the same study; can be conducted at relatively low cost; can easily be conducted in national samples; and can provide early insights about the potential for policies to change behavior. However, participants’ stated impressions of products do not perfectly predict actual behavior change [17,18]. 

By contrast, naturalistic laboratories that resemble real-world stores offer a novel solution for providing high-quality evidence about the effect of point-of-sale interventions on consumer behavior. Store laboratories can create a naturalistic, immersive experience that mimics real-life shopping experiences. These labs also allow researchers to measure real-life behavioral outcomes, which may have better construct validity than self-reported outcomes [19]. Moreover, store laboratories enable researchers to randomly assign participants to experience different interventions, allowing for strong causal inference without needing to rely on partnerships with retailers who may be resistant to implementing point-of-sale policies like taxes or warning labels. Studies have successfully used laboratory stores to examine point-of-sale interventions to reduce the purchase of sugary drinks [20], as well as tobacco [21,22] and alcohol [23]. However, studies have not yet examined the validity of using store laboratories as a proxy for real-world purchases, including evaluating 1) the extent to which behavior in store laboratories mirrors behavior in the world and 2) the extent to which participants perceive store laboratories to resemble real-world environments. Moreover, the feasibility of using store laboratories for longitudinal data collection remains to be established, especially among populations at risk for diet-related health disparities. 

To address the need for novel methods for testing point-of-sale nutrition policies and interventions, we conducted a validation and feasibility pilot study. The study took place in a naturalistic store laboratory and focused on parents who identify as Latino/a/x or Hispanic (hereinafter “Latinx”), given high rates of sugary drink consumption among Latinx parents and children [24,25,26]. We focused on sugary beverages because the consumption of sugary drinks is associated with health outcomes such as obesity and type 2 diabetes [27]. Thus, we aimed to assess whether beverage purchasing behavior in the store laboratory setting was similar to real-life beverage purchasing behavior. In addition, we aimed to examine whether participants viewed the store as being realistic and whether the protocol was feasible and acceptable. 

## 2. Materials and Methods

### 2.1. Store Development 

**Figure 1:**
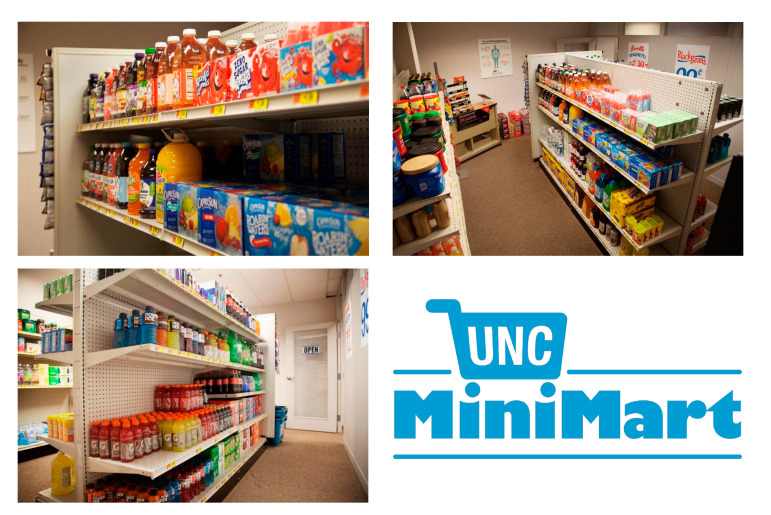
Photographs of and logo for UNC Mini Mart, a naturalistic store laboratory.

The research study was conducted at the UNC Mini Mart (Mini Mart), a naturalistic laboratory designed to mimic a real-life store (Figure 1). The Mini Mart measures 245 square feet and contains a commercial refrigerator, two gondola shelving units, and a register stand with a fully functioning point-of-sale payment system. For this study, the Mini Mart contained more than 200 unique packaged beverage products and approximately 60 unique food products and household goods. The process for selecting products and pricing the products is described in Supplementary Exhibit S1.

After initially stocking the Mini Mart, we invited ten Latinx community members to visit. The community members navigated the store to provide input on what products they felt were missing and how we could better organize the store to seem more realistic. We then added several new products and made additional organizational changes to the store layout based on their feedback. 

### 2.2. Study Protocol 

Participants attended five visits at the Mini Mart, spaced approximately one week apart (for a summary of each study visit, see Supplementary Table S2). At all visits, participants completed a shopping task, took an online survey, and received an incentive. At Visit 1, participants provided written informed consent. At Visits 1 and 3, participants provided beverage receipts from the prior week. At Visits 2–5, all sugary drinks were taxed (tax cohort) or labeled (warning cohort) (cohorts described below). At Visit 5, participants completed an exit interview after the online survey and received an informational handout on sugary drinks. The incentive for Visits 1–4 totaled USD 45 per visit and USD 70 for Visit 5. The incentive comprised of the total value of the items acquired from the Mini Mart plus the remaining value added to a Visa gift card. 

### 2.3. Receipt Collection

At Visits 1 and 3, participants were asked to provide all their grocery receipts from the prior week. This included supermarkets, department stores with groceries (e.g., Target), convenience stores, drug stores, and farmers’ markets, and did not include receipts from restaurants, fast-food establishments, or coffee shops. Additional details regarding receipt collection appear in Supplementary Exhibit S1. 

### 2.4. Shopping Task and Survey

At each visit, the research staff led a participant into the Mini Mart for a shopping task, instructing said participant to select a week’s supply of beverages for their household with a minimum of two beverages. Participants could also select food and household goods from the store. Children could accompany the participants in the Mini Mart. After completing the shopping task, the research assistant recorded the selected groceries using the point-of-sale system, notified the participant of the final cost, and bagged the groceries. The research staff then led the participant to a computer lab to complete a self-administered survey (programmed using Qualtrics) on either a computer or tablet. Participants took their food and beverages home, with the price deducted from their study incentive as described above. At Visits 2–5, participants were exposed to study stimuli in the store during their shopping task (Supplementary Figure S1). Additional details about study stimuli appear in Supplementary Exhibit S1). 

### 2.5. Measures 

The Visit 1 survey assessed standard demographic measures and beverage intake with items adapted from the Beverage Intake Questionnaire (BEVQ-15) [28]. The Visit 5 survey assessed reactions to study stimuli that are known to predict behavior change. The Visit 5 survey also assessed study acceptability using existing measures [29,30] and realism of the store using new items. Exact item wording appears in Supplementary Table S1. Several Visit 5 process measure responses were collected over the phone due to an error with survey programming. The survey could be completed in English or Spanish based on participants’ preferences.

### 2.6. Analysis 

For validation analyses, we calculated the percentage of participants that purchased each of the following beverage categories: (1) 100% fruit juice; (2) coffee and tea; (3) fruit drinks; (4) milk; (5) sports and energy drinks and flavored water; (6) soda; and (7) water in stores (i.e., according to receipts from the prior seven days before Visit 1) compared to in the Mini Mart during the Visit 1 shopping task. We then compared the percentages from real-world purchases vs. Mini Mart purchases for sugary drinks and non-sugary drinks using Chi-Square tests with Fisher’s *p*. We also reported the percentage agreement for purchasing any sugary drinks and purchasing any non-sugary drinks. We descriptively compared the medians of total volume in mL purchased from stores (i.e., according to receipts provided at Visit 1) and the total volume in mL purchased at the Mini Mart at Visit 1. For these calculations, we created a per capita/per day variable by dividing the volume in mL by household members and then dividing that number by seven. We reported results both including and excluding coffee and tea because these tend to be purchased in bulk and thus infrequently. We did not compare Visit 3 receipts with Mini Mart purchases because we did not expect them to be similar (participants were instructed to buy their beverages in the Mini Mart for that week). Analyses used Stata/SE version 16. 

## 3. Results 

Participants’ mean age was 36 years and nearly all were women (98%, Table 1). Most (84%) reported educational attainment of a high school diploma or less and 75% earned an annual household income of less than $25,000. Most (82%) completed the Visit 1 survey in Spanish and 72% spoke mostly or only Spanish at home. Over half (57%) reported a BMI in the overweight or obese categories. 

### 3.1. Validation 

**Figure 2:**
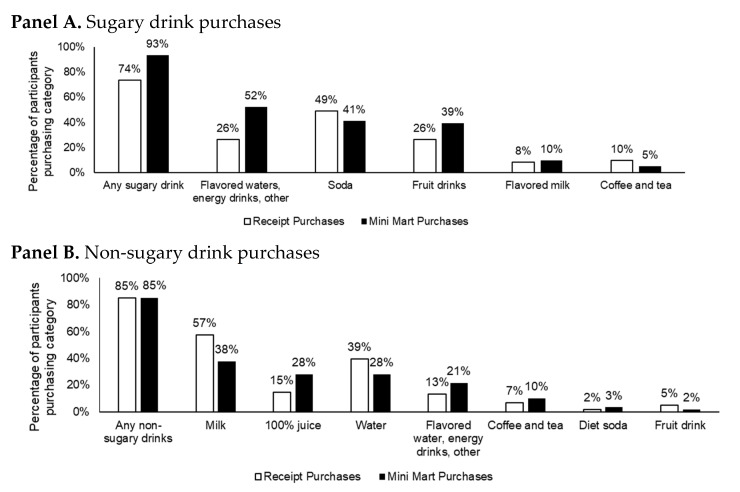
Percentage of participants purchasing each beverage type as measured via receipts. Receipts collected week prior to enrolling in the study and purchases in the UNC Mini Mart (at first study visit).

Overall, the percentage of parents who purchased sugary drinks in the Mini Mart (93%) was similar and not statistically significantly different from receipts (74%, *p* = 0.28), as well as non-sugary drinks (85% in the Mini Mart vs. 85% from receipts, *p* = 0.33). The percentage agreement was high for purchasing sugary drinks (74%) and non-sugary drinks (70%). The percentage of participants who purchased various beverages types is depicted in Figure 2. There was some variation within the subcategories of beverages. For example, 49% of people purchased soda according to receipt purchases compared to 41% in the Mini Mart, and 15% of people purchased 100% juice based on receipts compared to 28% in the Mini Mart. 

The median per capita volume of purchases (mL) per day and interquartile range (IQR) is presented in Supplementary Table S3. Comparing the total volume, parents purchased more beverages from stores (342 mL/capita/day, IQR 203–1238) than in the Mini Mart (319 mL/capita/day, IQR 176–476). Parents also purchased more non-sugary drinks from stores (245 mL/capita/day, IQR 110–686) than from the Mini Mart (137 mL/capita/day, IQR 18–269). Parents purchased fewer sugary drinks from stores (108 mL/capita/day, IQR 0–289) compared to the Mini Mart (152 mL/capita/day, IQR 74–276). 

**Figure 3:**
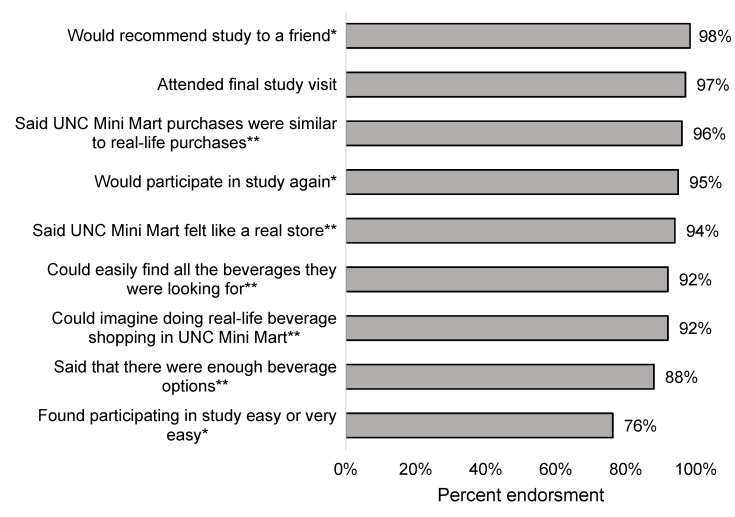
Realism of UNC Mini Mart and acceptability of participating in a study among Latinx parents (*n* = 61). * Responses collected via computer survey at Visit 5. ** Responses collected over the phone due to survey error.

Nearly all participants reported that their Mini Mart purchases were similar to real-life purchases (96%, Figure 3), that the Mini Mart felt like a real store (94%), and that they could find all the beverages they were looking for (92%). Moreover, nearly all participants reported that they could imagine completing their real-life beverage shopping in the Mini Mart (92%) and that there were enough beverage options (88%). 

### 3.2. Process Measures 

Participants found the study to be highly acceptable. Nearly all (98%) would recommend the study to a friend, 95% would participate in the study again, and 76% found participating in the study easy or very easy (Figure 3). Retention was very high, with 97% of participants attending the final study visit. Nearly all (92%) participants attended all five study visits, and 96% of total study visits were attended out of all possible visits. Reactions to stimuli appear in Supplementary Table S4. 

## 4. Discussion 

This study examined the validity and feasibility of using the Mini Mart, a naturalistic store laboratory, to evaluate the impact of point-of-sale policies and interventions on beverage purchases. We found that participants’ beverage purchasing patterns in the store laboratory were similar to their purchases in real stores. Moreover, the study protocol resulted in high retention and high acceptability. This realistic store laboratory holds promise for evaluating the impact of a variety of point-of-sale strategies on behavior change, including the effect of interventions on real-life purchasing behavior over time. 

Participants’ purchases from the receipt data collection and in the Mini Mart showed a high level of agreement in the percentage of parents who purchased sugary drinks. The proportion purchasing non-sugary drinks was also similar across the two settings. However, receipt data from stores showed a higher volume of overall beverage purchases, lower volume of sugary drink purchases, and higher volume of non-sugary drink purchases compared to the Mini Mart. One possibility for the observed differences is that participants may have purchased fewer beverages in the Mini Mart because the Mini Mart was located on the second floor of a building and only had shopping baskets, not carts, therefore limiting the volume participants could carry. In addition, although the study budget was designed to reflect typical beverage expenditures, the budget was set at an average based on top grocery retailers in North Carolina and might not reflect a given family’s shopping budget, especially given that the majority of participants reported an annual household income below $25,000. If the Mini Mart budget was larger than a parent’s typical budget, an income effect may have led parents to purchase products in the Mini Mart that they might not typically buy [31], potentially including more sugary drinks. Future studies could construct more tailored budgets based on a household’s typical expenditures [32]. A third possibility is that the receipt collection is itself an imperfect approach for assessing typical beverage purchases. It is possible that not all receipts were reported and the act of collecting receipts could have changed participants’ behavior. In addition, one week of receipt data may not be representative of a household’s “typical” purchases [33]. Receipt collection occurred the week prior to participants starting the study in the Mini Mart. This could have posed a particular problem for items purchased in bulk (e.g., multipacks of water); if parents purchased these products the week before, they may not have needed to purchase these items in the Mini Mart. In the future, it would be useful to validate Mini Mart results with multiple weeks of receipt data and collect these with a washout period of at least two weeks prior to beginning Mini Mart visits. 

In our study, we found that the store felt realistic to participants. Over 90% of participants stated that their purchases were similar to real-life, that the Mini Mart felt like a real store, and that they could imagine completing their grocery shopping there. Although 88% stated that there were enough beverage options, this is one area for potential improvement, as it is possible that the 12% who felt there were inadequate beverage options purchased less, resulting in the overall reduced beverage purchases we observed in the Mini Mart. One possibility would be to conduct a pre-experiment survey to identify the most-purchased products among participants and use those data to selectively stock the store with brands that are most frequently consumed by participants in the study. That said, randomized trials are internally valid even if participants’ purchases differ somewhat from real life because the observed differences across treatment groups can still be attributed to the treatment. 

The longitudinal study protocol was feasible and acceptable to participants. We recruited 61 Latinx parents to attend five study visits within the intended timeframe of three months. The ease of recruitment offers a promising sign for subsequent trials using similar methods, given that the main reason trials are terminated early is low enrollment [34]. All but two participants attended the final study visit, representing 97% retention, which is well above the typical threshold for acceptable retention of around 80% [35]. High retention in trials is important for both ensuring timely study completion and preventing potential biases caused by differential attrition by trial arm [35]. Nearly all (95% or more) participants said they would recommend the study to a friend and that they would participate in the study again if they had the opportunity. These acceptability ratings are similar to a tobacco study protocol [30] that was used successfully in two large-scale randomized trials [36,37]. 

There are advantages and disadvantages of utilizing a physical store laboratory compared to other approaches. Working directly with retailers would allow for a truly “real-world” setting for a trial, but collaborating with retailers presents challenges such as: finding retailers willing to participate in studies; obtaining transaction data; and randomizing at the individual level. In addition, food retailers may be reluctant to test interventions they view as potentially harmful to sales, such as taxes or warning labels. Another viable option is to conduct experiments in virtual 3D stores [38,39,40], which may facilitate faster and more affordable recruitment, although the simulated shopping environment is not as realistic as a physical store. Finally, some studies have used experimental online stores intended to replicate online grocery shopping or food ordering websites [41,42]. Online stores may also allow for faster recruitment and can be very realistic for people who typically buy groceries online; rates of online shopping have increased during the COVID-19 pandemic [43]. However, online stores would provide a less realistic experience for people who do not shop online.

Researchers should consider the relative advantages of different study settings alongside budget to determine the best path forward. Scientists who wish to use a physical store laboratory might consider partnering with colleagues studying different health issues (e.g., nutrition, tobacco, alcohol) and pooling resources to create a physical store laboratory that can be used in a variety of studies. The total amount of space required to create a store laboratory could be relatively small depending on the type of environment being emulated. For example, we were able to stock over 200 unique beverages, similar to beverage offerings in a grocery store, plus 60 additional food and household products in only 245 square feet of space. Finally, before conducting clinical trials in store laboratories, researchers should consider budgeting for pilot studies to develop and test protocols for: (1) managing inventory throughout the study; (2) exposing participants to study stimuli; (3) randomizing participants to study arms; (4) providing shopping instructions to participants; (5) tracking participants’ selections; and (6) collecting and administering payment exchanges.

The use of a novel, realistic store laboratory resembling a real store was one strength of this study. We also successfully recruited and retained a sample of Latinx adults, an important population for nutrition research that is often underrepresented in research studies [44,45]. However, the generalizability of our findings to other populations, locations, and product types remains to be established. Finally, the small sample size and non-randomized design precluded us from examining the effects of tax and labeling policies, which were beyond the scope of this feasibility study. 

## 5. Conclusions 

Many experiments evaluating policies and interventions have been conducted using artificial exposure and settings, which can mask the complexity of real-world decision making in which consumers must evaluate many products and factors (e.g., price, marketing, quality, brand preferences) quickly and at once. Our naturalistic store laboratory addresses these limitations by improving the realism of point-of-sale studies, while still allowing for experimental control. This model could be leveraged to evaluate the impact of a variety of public health strategies (e.g., excise taxes, manipulating product placement) on the healthfulness of food purchases. 

## Figures and Tables

**Table 1:** Characteristics of study participants in a naturalistic store laboratory.

	*n*	%
Cohort		
Tax	31	51%
Warning	30	49%
Age		
18–29 years	11	18%
30–39 years	31	51%
40–49 years	17	28%
50+ years	2	3%
Mean in years (SD)	36.3	7.3
Gender		
Man	1	2%
Woman	57	98%
Educational attainment		
Less than high school or GED	22	39%
High school diploma or GED	29	51%
Four-year college degree	5	9%
Master’s degree or greater	1	2%
State of health		
Excellent, very good, or good	33	54%
Fair or poor	28	46%
Preferred language to speak at home		
Mostly or only English	4	7%
Mostly or only Spanish	42	72%
Equally Spanish and English	12	21%
Household income, annual		
USD 0–24,999	46	75%
USD 25,000+	15	25%
Number of children in household (age 0–18)		
One	13	21%
Two	32	52%
Three or more	16	26%
Used SNAP in the last year	20	33%
Used WIC in the last year	17	28%
Average weekly spending on beverages		
Less than USD 5	3	5%
USD 5–10	9	15%
USD 11–15	11	18%
USD 16–20	15	25%
USD 21–25	13	21%
More than USD 25	10	16%
Body mass index (BMI, kg/m^2^)		
Underweight (<18.5)	2	3%
Healthy weight (18.5–24.9)	9	15%
Overweight (25.0–29.9)	12	20%
Obese (>29.9)	23	38%
Missing	15	25%
Mean BMI (SD)	30.9	9.6
Language of Survey		
English	11	18%
Spanish	50	82%

Missing demographic data ranged from 0% to 7%. Demographic characteristics did not differ between the two cohorts, except for number of children in household (*p* < 0.05). SNAP = Supplemental Nutrition Assistance Program. WIC= Special Supplemental Nutrition Program for Women, Infants, and Children.

## Data Availability

The data presented in this study are available on request from the corresponding author.

## Supplementary Materials

The following are available online at https://www.mdpi.com/article/10.3390/ijerph18168764/s1, Table S1: Measures used in study in the UNC Mini Mart, a naturalistic store laboratory. Measures assessed at Visit 5, Table S2: Protocol elements for study in UNC Mini Mart, a naturalistic store laboratory, Table S3: Total volume of beverages purchased, measured via receipts at baseline visit compared to purchases in UNC Mini Mart at Visit 1(mL/capita/day), Table S4: Reactions to sugary drink price increases and warning labels in the UNC Mini Mart, a naturalistic convenience store lab, Figure S1: Stimuli used the UNC Mini Mart, a naturalistic convenience store lab, Exhibit S1: Additional methodological details about product selection, pricing, and receipt data entry.
