# Supplementary material for: Using a Naturalistic Store Laboratory for Clinical Trials of Point-of-Sale Nutrition Policies and Interventions: A Feasibility and Validation Study
Source: Int J Environ Res Public Health. 2021 Aug 19;18(16):8764. doi: 10.3390/ijerph18168764 (PMC8394834; doi:10.3390/ijerph18168764)
Supplement: Supplementary file 1 [file ijerph-18-08764-s001.zip › ijerph-1323373-supplementary.pdf]

Supplementary Table S1. Measures used in study in the UNC Mini Mart, a naturalistic store laboratory. Measures assessed at Visit 5.

**Question (English)**	**Response Options (English)**	**Question (Spanish)**	**Response Options (Spanish)**
How much did the [labels / price increases] grab your attention?	1=Not at all<br>2=A little<br>3=Somewhat<br>4=Quite a bit<br>5=Very much	¿Llamaron su atención estas [etiquetas/ subidas de precio]?	1=No<br>2=Un poco<br>3=Regular<br>4=Bastante<br>5=Mucho
How often did you notice the [labels/price increases]?	1=Never<br>2=Rarely<br>3=Sometimes<br>4=Often<br>5=All the time	¿Con qué frecuencia notó las [etiquetas/subidas de precio]?	1=Nunca<br>2=Raramente<br>3=Algunas veces<br>4=A menudo<br>5=Todo el tiempo
How often did you read or look closely at the labels?	1=Never<br>2=Rarely<br>3=Sometimes<br>4=Often	¿Con qué frecuencia las leyó o miró con más atención?	1=Nunca<br>2=Raramente<br>3=Algunas veces<br>4=A menudo

	5=All the time		5=Todo el tiempo
How often did you notice the price increases?	1=Never<br>2=Rarely<br>3=Sometimes<br>4=Often<br>5=All the time	¿Con qué frecuencia leyó o miró con más atención los precios?	1=Nunca<br>2=Raramente<br>3=Algunas veces<br>4=A menudo<br>5=Todo el tiempo
How believable were the [labels/prices]?	1=Not at all<br>2=A little<br>3=Somewhat<br>4=Quite a bit<br>5=Very much	¿Parecen reales [estas etiquetas/los precios]?	1=No<br>2=Un poco<br>3=Regular<br>4=Bastante<br>5=Mucho
How much did the [labels/price increases] make you think about the health problems caused by drinking beverages with added sugar?	1=Not at all<br>2=A little bit<br>3=Some<br>4=Quite a bit<br>5=Very much	¿[Las etiquetas/las subidas de precio] le hacen pensar en los problemas de salud que causan las bebidas azucaradas?	1=No<br>2=Un poco<br>3=Algo<br>4=Bastante<br>5=Mucho

Who did you talk to about the [labels/price increases]? (Check all that apply)	1=My spouse or significant other<br>2=My child<br>3=Other family member<br>4=Friend<br>5=Co-worker<br>6=Health care provider<br>7=Someone I did not previously know<br>8=Other<br>9=I did not talk to anyone about the labels	¿Con quién habló de las [etiquetas/subidas de precio]? (marque todo lo que corresponda)	1=Mi cónyuge o pareja<br>2=Mi hijo o hija<br>3=Otro familiar<br>4=Un amigo<br>5=Un compañero de trabajo<br>6=Un profesional médico<br>7=Alguien que no conocía antes<br>8=Otro<br>9=No hablé con nadie sobre las etiquetas
How much did the [labels on the beverages/price increases] make you feel anxious?	1=Not at all<br>2=A little<br>3=Somewhat<br>4=Very<br>5=Extremely	¿[Las etiquetas en las bebidas/las subidas de precio] le hicieron sentir ansiedad?	1=No<br>2=Un poco<br>3=Regular<br>4=Mucho<br>5=Muchísimo

How much did the [labels on the beverages/price increases] make you feel guilty?	1=Not at all<br>2=A little<br>3=Somewhat<br>4=Very<br>5=Extremely	¿[Las etiquetas en las bebidas/las subidas de precio] le hicieron sentir culpabilidad?	1=No<br>2=Un poco<br>3=Regular<br>4=Mucho<br>5=Muchísimo
Would you recommend this study to a friend?	1=Definitely not<br>2=Probably not<br>3=Maybe<br>4=Probably yes<br>5=Definitely yes	¿Recomendaría este estudio a un amigo o amiga?	1=Definitivamente no<br>2=Seguramente no<br>3=Quizás<br>4=Seguramente sí<br>5=Definitivamente sí
The beverages I purchased in this store were similar to my	1=Strongly disagree<br>2=Somewhat disagree<br>3=Neither agree nor disagree	Las bebidas que compré en esta tienda son parecidas a las que compro normalmente.	1=Completamente en desacuerdo<br>2=Algo en desacuerdo<br>3=Ni de acuerdo ni en desacuerdo<br>4=Algo de acuerdo

regular beverage purchases.	4=Somewhat agree<br>5=Strongly agree		5=Completamente de acuerdo
If you had the chance, would you participate in this study again?	1=Definitely not<br>2=Probably not<br>3=Maybe<br>4=Probably yes<br>5=Definitely yes	¿Participaría en este estudio de nuevo si le dieran la oportunidad?	1=Definitivamente no<br>2=Seguramente no<br>3=Quizás<br>4=Seguramente sí<br>5= Definitivamente sí
This store felt like a real store.	1=Strongly disagree<br>2=Somewhat disagree<br>3=Neither agree nor disagree<br>4=Somewhat agree<br>5=Strongly agree	La tienda parecía de verdad.	1=Completamente en desacuerdo<br>2=Algo en desacuerdo<br>3=Ni de acuerdo ni en desacuerdo<br>4=Algo de acuerdo<br>5=Completamente de acuerdo
I could easily find all the beverages I was looking for in the store.	1=Strongly disagree<br>2=Somewhat disagree<br>3=Neither agree nor disagree<br>4=Somewhat agree	En la tienda encontré todas las bebidas que buscaba con facilidad.	1=Completamente en desacuerdo<br>2=Algo en desacuerdo<br>3=Ni de acuerdo ni en desacuerdo<br>4=Algo de acuerdo<br>5=Completamente de acuerdo

	5=Strongly agree		
I was able to imagine doing my real-life beverage shopping in the store.	1=Strongly disagree<br>2=Somewhat disagree<br>3=Neither agree nor disagree<br>4=Somewhat agree<br>5=Strongly agree	Me era posible imaginar que hacía mis compras de bebidas de verdad.	1=Completamente en desacuerdo<br>2=Algo en desacuerdo<br>3=Ni de acuerdo ni en desacuerdo<br>4=Algo de acuerdo<br>5=Completamente de acuerdo
There were enough beverage options in the store.	1=Strongly disagree<br>2=Somewhat disagree<br>3=Neither agree nor disagree<br>4=Somewhat agree<br>5=Strongly agree	Había suficientes opciones de bebidas.	1=Completamente en desacuerdo<br>2=Algo en desacuerdo<br>3=Ni de acuerdo ni en desacuerdo<br>4=Algo de acuerdo<br>5=Completamente de acuerdo
Overall, how difficult or easy was it to be in this study?	1=Very difficult<br>2=Difficult<br>3=Neither difficult nor easy<br>4=Easy	En general, ¿fue fácil o difícil participar en este estudio?	1=Muy difícil<br>2=Difícil<br>3=Regular<br>4=Fácil<br>5=Muy fácil

	5=Very Easy		

Supplementary Table S2. Protocol elements for study in UNC Mini Mart, a naturalistic store laboratory

	Informed Consent	Beverage receipts from prior week recorded by study staff	Exposed to stimuli in UNC Mini Mart	Shopping task	Post-shopping survey	Exit Interview
Visit 1	X	X		X	X	
Visit 2			X	X	X	
Visit 3		X	X	X	X	
Visit 4			X	X	X	
Visit 5			X	X	X	X

Supplementary Table S3. Total volume of beverages purchased, measured via receipts at baseline visit compared to purchases in UNC Mini Mart at Visit 1 (mL/capita/day)

	Receipts (*n*=59)		UNC Mini Mart (*n*=59)	
**Excluding coffee and tea**	Median	IQR[a]	Median	IQR[a]
Total	342	203-1238	319	176-476
Sugary drinks	108	0-289	152	74-276
Non-sugary drinks	245	110-686	137	18-269
**Including coffee and tea**				
Total	480	217-1253	333	231-593
Sugary drinks	108	0-405	153	76-306
Non-sugary drinks	257	137-709	165	55-306

[a] IQR=interquartile range

Supplementary Table S4. Reactions to sugary drink price increases and warning labels in the UNC Mini Mart, a naturalistic convenience store lab

	Tax cohort (*n*=30)	Warning cohort (*n*=29)
Price increases/labels grabbed their attention [a]	38%	59%
Noticed the price increases/labels frequently [b]	52%	72%
Read or looked closely at the price tag/labels [b]	66%	59%
Found the prices/labels believable [a]	62%	69%
Price increases/labels made them think about sugary drink health problems [a]	59%	79%
Talked to someone about the price increases/labels	69%	66%
Felt anxious because of the price increases/labels [c]	25%	39%
Felt guilty because of the price increases/labels [c]	18%	54%

Note. Denominator ranged from 28 to 29 in Cohort 1 and 28 to 29 in Cohort 2 based on varying amounts of missing data. All data from Visit 5 survey.

[a] Responded "Somewhat," "Quite a bit," or "Very much" rather than "Not at all" or "A little"

[b] Responded "Sometimes," "Often," or "All the time" rather than "Never" or "Rarely"

[c] Responded "Somewhat," "Very," or "Extremely" rather than "Not at all" or "A little"

Supplementary Figure S1. Stimuli used in study in the UNC Mini Mart, a naturalistic convenience store lab

Panel A. Tax cohort example

Before implementing tax

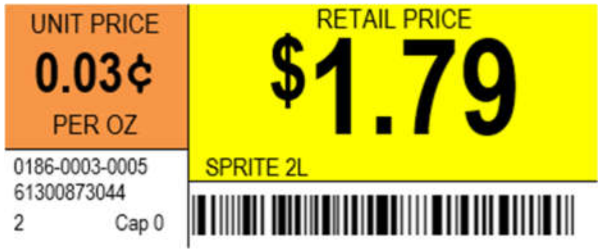

After implementing tax

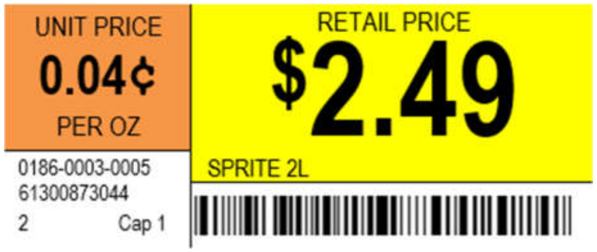

Panel B. Health warning cohort label

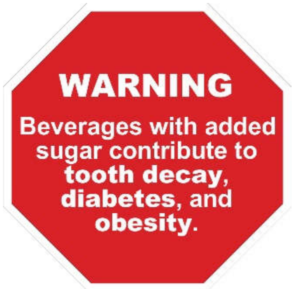

Supplementary Exhibit S1. Additional methodological details about product selection, pricing, and receipt data entry

Product selection. To ensure the beverages offered in the store provided an accurate representation of popular beverages in the area, we used Nielsen Homescan data on 2014 household beverage purchases in NC. There were 15 beverage categories included in the dataset (e.g., soft drinks, diet soft drinks, plain water). Within each beverage category, we created a list of beverages to stock based on the beverage products that had the highest sale volume (top 15 products) and beverages that had the most units purchased (top 15 products) –there was often overlap between sales volume and units purchased. These products were then compared to the top products purchased in Latinx households in NC, per Nielsen Homescan data. Any products that were unique to the list of top beverages among Latinx households were added to the list of beverages to stock. Additionally, we stocked at least one generic brand for every beverage category, as well as several additional beverages. These additional beverages were determined based on consultation with members of the target population and our team of nutritionists to ensure there was a wide variety of sugary drinks and non-sugary drinks within the beverage categories.

To select which food and household goods to stock in the Mini Mart, research staff visited five convenience stores in Chapel Hill, NC to document which product categories were commonly sold. Within the categories of food and household goods, the research staff determined approximate distributions of the goods (e.g., about 20% of household goods sold were cleaning products). We then reviewed Euromonitor data[48] to identify popular brands for each product category. Finally, we reviewed the food items we planned to stock to ensure the Mini Mart was SNAP compliant.

Study stimuli. We assigned participants to two cohorts (tax vs. warning) that saw different study stimuli. Cohorts were assigned by date, with the tax cohort enrolling in September 2019 and the warning cohort enrolling in October 2019. Participants in the tax cohort were exposed to a price increase on all sugary drinks (i.e., all drinks with added sugar except for dairy and dairy-substitute products). The prices were increased by $0.01 per ounce or fluid ounce, in line with the sugary drink taxes passed in Albany, NY, Berkeley, CA, Oakland, CA, and Cook County, IL, with the final price rounded to the nearest $0.09. Participants in the warning cohort were exposed to a warning label on all sugary drinks that read "WARNING: Beverages with added sugar contribute to tooth decay, diabetes, and obesity". The language was modeled after proposals in several US states [26] and has been previously shown to discourage sugary drink purchases [15].

Prices. To set prices for the beverages, we used Nielsen Homescan data[47] to determine the top two grocery retailers in NC. Through referencing the websites of the top two retailers, as well as visiting their local stores, research staff documented the regular prices and the sale prices for all beverage products stocked in the Mini Mart. Once all prices were documented, we averaged the non-sale prices of each beverage from the two stores. Once all average prices were calculated, they were rounded to the nearest $0.09 to create uniformity in the store. To price the food and household goods, research staff visited a convenience store nearby the Mini Mart store and used the corresponding prices. For non-beverage products that were in the Mini Mart but not sold at the convenience store, we used the product's price from the top retailer.

Receipt data entry. For the receipt data collection, prior to the shopping task, a research assistant reviewed each receipt with the participant while entering all relevant information about each beverage into a computer survey programmed using Qualtrics. Relevant

information included the receipt date, store name, beverage brand and flavor, whether the beverage was ready to drink, the total beverage volume, total units purchased, and total price of the beverage. Additionally, the research assistant noted if anyone else in the participant's household had purchased beverages in the week prior. After reviewing all receipts, the research assistant asked the participants if there were any other packaged beverages they had purchased in the previous week for which they did not have receipts. Research staff then verbally obtained the same information about these beverages and entered this information into the survey.
